# Intermittent Ovarian Torsion in Pregnancy

**DOI:** 10.5811/cpcem.2016.12.32932

**Published:** 2017-03-15

**Authors:** Randall Young, Kelly Cork

**Affiliations:** * Kaiser Permanente San Diego Medical Center, Department of Emergency Medicine, San Diego, California; † Kaiser Permanente San Diego Medical Center, Department of Obstetrics and Gynecology, San Diego, California

## Abstract

Ovarian torsion during pregnancy is a fairly uncommon complication with a high patient morbidity and fetal mortality if not immediately treated. Ovarian torsion should be considered a clinical diagnosis, and a high level of clinical suspicion is needed by the practitioner to ensure that this diagnosis is not missed. We present an unusual case of intermittent ovarian torsion discussing both the presentation and the operative and post-operative management.

## INTRODUCTION

Ovarian torsion is a disorder with a very high patient morbidity. When the patient is pregnant this can also lead to fetal mortality and potential loss of fertility for the patient. The presentation of ovarian torsion can mimic many other intraabdominal pathologic conditions. It requires a high level of clinical suspicion from the provider to not miss this potentially devastating diagnosis. Imaging and laboratory results may be used as support; however, the diagnosis should primarily be made on a strong history and physical exam. When the physical examination is not congruent with the level of patient distress, ischemic pain from an ovarian torsion must be considered.

## CASE REPORT

The patient was a 34-year-old gravid 1 para 0 female at approximately 10 weeks gestational age by last menstrual period who presented to a community emergency department (ED) with complaints of right lower quadrant abdominal pain. The patient reported that the pain woke her up from sleep at 3 a.m. The patient arrived to the ED approximately three hours after the onset of pain. The patient described the pain as a “constant pinching” localized only in the right lower quadrant and it was unrelieved by acetaminophen. The patient denied nausea or vomiting. She also denied any vaginal bleeding or discharge.

A physical exam revealed a temperature of 36.8 Celsius, pulse of 82 beats per minute (BPM), respiratory rate of 18 breaths per minute, and blood pressure of 125/67 mmHg. At the time of the initial examination the patient appeared comfortable and in no acute distress. Her abdominal exam revealed a soft abdomen with normal bowel sounds. No masses, distention or tenderness were detected. Pelvic exam was conducted and was noted to have no vaginal discharge or bleeding, and no pelvic masses were appreciated by the provider. However, it was noted that body habitus limited the exam.

A formalized pelvic ultrasound (US) showed a single live intrauterine pregnancy with a fetal heart rate of 163 BPM. Large right ovarian cysts were noted and there was arterial flow noted centrally in both the left and right ovaries. The right ovary measured 7.7 cm × 4.8 cm × 5.9 cm, whereas the left ovary was only 1.9 cm × 1.4 cm × 1.6 cm.

The case was discussed with the on-call obstetrician who stated that it was most likely a corpus luteal cyst and the pain should resolve on its own. Since the pain had completely, resolved the patient was discharged home with scheduled follow-up in two days with obstetrics and gynecology (OB/GYN).

The patient had an unplanned return to the ED nine hours from time of discharge with recurrence of her abdominal pain. The patient reported that the pain was located in the same location and felt similar, although now it was much more intense and was not resolving. She was also very nauseated and actively vomiting.

Reexamination revealed a temperature of 36.1 degrees Celsius, pulse rate of 64 BPM, respiratory rate of 18 breaths per minute and a blood pressure of 133/78 mmHg. At the time of reevaluation the patient appeared in acute distress, doubled over and moaning in pain. The patient was intermittently vomiting what appeared to be gastric contents. However, her abdominal exam again showed no peritoneal signs, no focal tenderness and no masses. The patient’s abdominal examination did not match her level of distress. OB was again consulted and requested an additional formal US.

This time, repeat US again showed an enlarged right ovary with multiple cysts. At the time of the repeat US the right ovary measured 8.23 cm × 8.41 cm × 5 cm, whereas the left ovary was 2.22 cm × 2.94 cm × 3.22 cm. Using color Doppler, blood flow was not demonstrated in the right ovary, whereas left ovary demonstrated adequate blood flow.

OB evaluated this patient and took her to the operating room. The patient had an exploratory laparoscopy performed, which revealed that the right infundibulopelvic (IP) ligament was twisted times three. A laparoscopic needle and syringe were used to drain two simple ovarian cysts. The right ovary was manually detorsed and healthy viable ovarian tissue returned. Postoperatively the patient was started on intravaginal progesterone 200mg for the following four weeks. At term the patient had a spontaneous vaginal delivery of a healthy girl.

## DISCUSSION

Ovarian torsion during pregnancy is a fairly uncommon complication with a high patient morbidity and fetal mortality if not immediately treated.^1^ Torsion more commonly occurs on the right rather than the left with an incidence of 3:2.^1^ Ovarian torsion rises fivefold in pregnancy to approximately five in 10,000. This typically occurs between the sixth and fourteenth weeks of gestation. The most common cause is the growth of a corpus luteal cyst, which usually spontaneously regresses by the second trimester. The reason that ovarian torsion is thought to occur more commonly on the right rather than the left is because it is believed that the sigmoid colon limits the mobility of the left ovary.^2^ If the IP ligament is successfully detorsed, it is common practice to excise or drain the cysts that are believed to have caused the torsion to prevent repeat torsion. Since the typical cyst in the first trimester is a corpus luteal cyst, which supports the pregnancy until the placenta is adequately developed at the end of the first trimester, drainage or excision of the cyst could result in pregnancy loss. Therefore, some obstetricians opt to treat with supplemental progesterone to support the pregnancy until the placenta can be further developed, usually in the second trimester.

## CONCLUSION

The diagnosis of ovarian torsion must be made clinically. As demonstrated in this case, a normal Doppler ultrasound does not exclude intermittent ovarian torsion. Torsion must be considered in any female with sudden onset, severe lower abdominal pain. As in this case, the abdominal examination often does not correspond with the level of distress that the patient would present with. When torsion is considered, early consultation with OB/GYN should be obtained as both current and future fertility are at stake. Successful detorsion must be performed in a timely manner to protect fertility.

## Figures and Tables

**Image 1:**
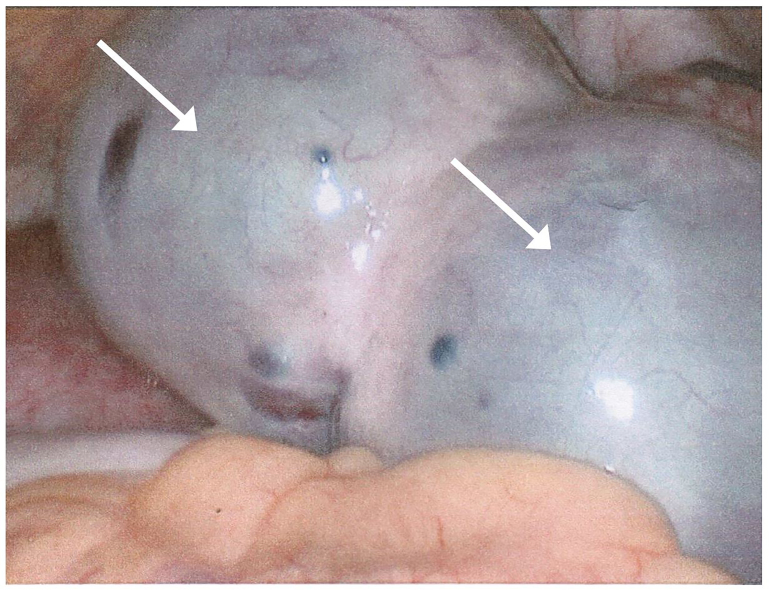
Intraoperative laparoscopic view of two large ovarian cysts with ischemic discoloration of the tissue.

**Image 2:**
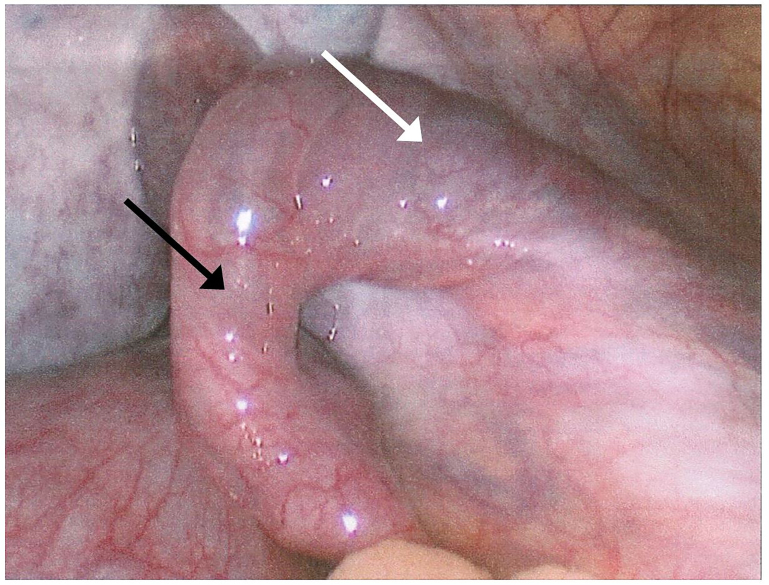
Intraoperative laparoscopic view status post drainage of cysts (white arrow) and detorsion of infundibulopelvic ligament (black arrow); note return of healthy pink tissue.

## References

[1] Lentz GM, Lobo RA, Gershenson D (2012). Comprehensive Gynecology.

[2] Sasaki KJ, Miller CE (2014). Adnexal torsion: review of the literature. J Minim Invasive Gynecol.

